# Ganoderma Microsporum Immunomodulatory Protein Alleviates Inflammaging and Oxidative Stress in Diabetes-Associated Periodontitis via Nrf2 Signaling Activation: An In Vitro Study

**DOI:** 10.3390/antiox13070817

**Published:** 2024-07-08

**Authors:** Ni-Yu Su, Min Yee Ng, Heng-Yi Liao, Yi-Wen Liao, Movina Wu, Shih-Chi Chao, Cheng-Chia Yu, Yu-Chao Chang

**Affiliations:** 1 School of Dentistry, Chung Shan Medical University, Taichung 40201, Taiwan; freulich2002@gmail.com (N.-Y.S.); ngminyee_92@hotmail.com (M.Y.N.);; 2 Department of Dentistry, Chung Shan Medical University Hospital, Taichung 40201, Taiwan; 3 Institute of Oral Sciences, Chung Shan Medical University, Taichung 40201, Taiwan; rabbity0225@gmail.com (Y.-W.L.);; 4 Department of Medical Research, Chung Shan Medical University Hospital, Taichung 40201, Taiwan

**Keywords:** diabetic periodontitis, Ganoderma immunomodulatory protein

## Abstract

Periodontitis, characterized by inflammation and loss of periodontal tissue, is a significant health complication for individuals with diabetes mellitus (DM). Buildup of advanced glycation end-products (AGEs) in DM poses an increased risk of periodontitis via inflammaging. Ganoderma immunomodulatory protein (GMI) shows promise in suppressing inflammaging by mitigating oxidative stress and inflammation via Nrf2 modulation. However, its specific protective effects are not fully understood. Thus, this study aimed to investigate GMI’s anti-inflammaging properties and its underlying mechanism in diabetic-associated periodontitis (DP). We first simulated DP by culturing human gingival fibroblasts (HGFs) with AGEs and lipopolysaccharides from *P. gingivalis* (LPS). We then evaluated the impact of GMI on cell proliferation, migration and wound healing. Additionally, we assessed GMI’s effects on the components of inflammaging such as reactive oxygen species (ROS) formation, cellular senescence expression, IL-6 and IL-8 secretions, and NF-κB phosphorylation. Next, we explored whether GMI’s anti-inflammaging effects are mediated through the Nrf2 pathway by evaluating Nrf2 and HO-1, followed by the assessment of IL-6 and IL-8 post-Nrf2 knockdown. Our findings revealed that GMI treatment suppressed ROS production, cell senescence, IL-6 and IL-8 and NF-κB phosphorylation. Furthermore, GMI upregulated Nrf2/HO-1 expression and its protective effects were reversed when Nrf2 was knocked down. In conclusion, GMI exerts its anti-inflammaging effect via the modulation of the Nrf2/NF-κB signaling axis in DP in vitro, highlighting its potential as an effective adjunct treatment for diabetes-related periodontitis.

## 1. Introduction

Periodontitis, a local inflammatory disease of the periodontium, is closely associated with the accumulation of dental plaque, providing a habitat for the oral pathogenic bacteria to thrive in and elicit host immune response. If left uncontrolled, the host immune response towards these oral pathogens often leads to inflammation and subsequent destruction of the tooth-supporting structures, such as alveolar bone and periodontal ligaments [1]. This local disease exhibits a bidirectional relationship with diabetes mellitus (DM), wherein individuals with DM have heightened inflammatory responses to periodontal microbes, while periodontal health significantly impacts the metabolic control in DM patients [2,3,4]. It is acknowledged that the local inflammatory responses lead to elevated systemic inflammation, which subsequently causes insulin resistance and upregulated blood sugar levels, exacerbating DM [5,6]. Thus, it is crucial to treat the heightened immune response in periodontal tissue in order to curb the vicious cycle between periodontitis and DM. 

Growing evidence shows that the accumulation of advanced glycation end-products (AGEs) in hyperglycemic patients contributes to inflammaging, which creates a heightened immune response [7]. Inflammaging, a term coined to describe the low-grade chronic inflammatory state associated with aging, involves a complex interplay of various mechanisms, with the key players being (1) the upregulation of cellular senescence, (2) senescence-associated secretory phenotype and (3) oxidative stress [8,9,10,11,12]. Understanding the involvement of AGEs in inflammaging sheds light on potential therapeutic targets for mitigating DM-associated complications, including diabetic periodontitis (DP). 

Notably, AGEs are recognized for their ability to prompt cellular senescence, a condition marked by irreversible growth arrest. A recent study has shown that gingival tissue from the DP group exhibits higher levels of senescence markers compared to tissue from those with periodontitis alone [13]. Senescent cells have the ability to release various inflammatory cytokines like interleukin-6 (IL-6) and interleukin-8 (IL-8), collectively known as the senescence-associated secretory phenotype (SASP) [14]. Significantly elevated circulating levels of SASP have been observed in the DP group compared to those with periodontitis only, with these SASP levels positively correlating with clinical parameters [13]. Aside from senescent cells and SASP secretions, AGEs also induce the formation of reactive oxygen species (ROS) and cellular oxidative stress through lipid peroxidation and glycoxidation [15,16,17]. These mechanisms collectively contribute to a hyperinflammatory immune response [15,16,18] and are associated with exacerbating periodontal tissue destruction in DP [19,20]. 

The current gold standard for DP involves mechanically removing bacteria biofilm and calculus through scaling and root planing (SRP). However, SRP has limitations, particularly in cleaning deep pockets effectively, which may hinder its ability to fully resolve DP. Moreover, this approach focuses on reducing bacterial load but does not address the hyperinflammatory host reaction in DP. Thus, finding an alternative treatment targeting inflammaging is essential in alleviating DP.

Few studies have underscored the importance of the Nrf2 axis in regulating inflammaging or DM-associated complications [21]. Therefore, we employed a natural compound known to target the Nrf2 axis in this study: Lingzhi (Ganoderma lucidum). This venerable woody mushroom has been traditionally used to extend lifespan, enhance vigor and vitality, and improve immune responses in cancer patients [22]. Lingzhi is recognized for its remarkable antioxidant and anti-inflammatory traits through the modulation of the Nrf2 signaling pathway [23,24]. The therapeutic benefits of its components, such as triterpenoids, polysaccharides, and fungal immunomodulatory proteins (FIPs), have been well documented [25]. Among these, GMI, a FIP derived from Ganoderma microsporum, has shown potential in resolving oxidative stress or inflammation in various cells such as human alveolar epithelial A549 cells [26], microglial cells [27] and fibrotic buccal mucosal fibroblasts [28]. However, little is known about its effects on DP, particularly on inflammaging. Hence, this study aimed to explore the anti-inflammaging properties of GMI in DP and elucidate its underlying mechanism. We hypothesized that GMI could suppress inflammaging in DP through the activation of the Nrf2 signaling pathway.

## 2. Materials and Methods

### 2.1. Human Gingival Fibroblasts (HGFs) Cell Culture

Recombinant GMI protein was synthesized and provided by MycoMagic Biotechnology (New Taipei, Taiwan). The culture of human gingival fibroblasts (HGFs) adhered to the protocols approved by the Institutional Review Board of Chung Shan Medical University Hospital (CSMUH No.: CS2-22045). HGFs were obtained from crown lengthening procedures performed on two healthy individuals, using the explant technique as previously outlined [29,30]. Cell cultures utilized in this study ranged from the third to eighth passages. To assess the impact of GMI, HGFs were exposed to varying concentrations of GMI for 24 h after stimulation with AGEs (200 μg/mL) and LPS (10 μg/mL). All experiments were conducted using HGFs sourced from two independent samples. AGEs-BSA was procured from BioVision (Milpitas, CA, USA).

### 2.2. Cell Viability

Cells were plated onto 96-well plates (Costar, Corning Inc., Rochester, NY, USA) at a density of 1 × 10^4^ cells per well and were stimulated with AGEs and LPS. GMI treatment of various dosages were given at the 24th hour, and the cells were further cultured at 37 °C for 48 h. Cell viability was assessed using the MTT [3-(4,5-dimethylthiazol-2-yl)-2,5-diphenyl tetrazolium bromide] assay according to the manufacturer’s instructions. The absorbance at 570 nm for the control group (0 μg/mL GMI) was designated as 100%, and the data were expressed as a percentage of the control.

### 2.3. Measurement of ROS Generation

Flow cytometry was utilized to assess the production of ROS using the fluorescence of 2′,7′-dichlorofluorescein (DCF), which is the oxidation product of 2′,7′-dichlorodihydrofluorescein diacetate (DCFH-DA; Sigma-Aldrich, Madrid, Spain). DCFH-DA is known for its sensitivity to H_2_O_2_/NO-based radicals and O^−2^. HGFs were treated with 10 μM DCFH-DA for 60 min at 37 °C, followed by two washes with PBS. Flow cytometry (Becton–Dickinson, CA, USA) was employed to measure the DCF fluorescence of 1 × 10^4^ cells, with excitation and emission wavelengths set at 488 and 525 nm, respectively [29].

### 2.4. Senescence-Associated Beta-Galactosidase (SA-β-Gal) Staining

HGFs were seeded into 6-well plates. After experimental conditions and washings with PBS, the plates were incubated with a fixative solution for galactosidase staining for 20 min at room temperature. Afterward, the cells were rinsed three times with PBS. Following this, the plates were incubated with a working solution overnight at 37 °C. Finally, the plates were examined using an optical microscope, and quantitative analysis was performed on three separate microscope fields.

### 2.5. Cell Migration Assay

The migration capacities were assessed using a 24-well plate Transwell system equipped with a polycarbonate filter membrane featuring an 8 μm pore size (Corning, Flintshire, UK). Cell suspensions were seeded into the upper compartment at a density of 1 × 10^5^ cells per well in serum-free medium. Following 24 h, the filter membrane was fixed for 1 h, and cells that had migrated to the serum-containing media in the bottom chamber were stained with crystal violet (Sigma-Aldrich). Subsequently, these cells were counted from five different visual areas under a microscope at 100-fold magnification.

### 2.6. Wound-Healing Assay

After seeding cells into a 12-well culture dish, the cells were cultured until they reached approximately 80% confluence. Subsequently, a wound was created in the monolayer by scratching across the center of the well using a sterile 200 μL pipette tip. The cells were then allowed to grow for an additional 48 h before being stained with crystal violet. At both 0 and 48 h post-scratching, the migration of cells towards the wound site was measured using a microscope.

### 2.7. Quantitative Reverse-Transcription Polymerase Chain Reaction (RT-PCR)

Total RNA was isolated from cells using Trizol reagent (Invitrogen Life Technologies, Carlsbad, CA, USA) following the manufacturer’s instructions. The isolated RNA was then reverse transcribed using the Invitrogen Life Technologies Superscript III first-strand synthesis system. PCR amplification was conducted with an initial denaturation step at 95 °C for 1 min, followed by 35 cycles of denaturation at 95 °C for 15 s, annealing at 55 °C for 15 s, and extension at 72 °C for 30 s. SYBR Green-based quantitative real-time PCR (qRT-PCR) experiments were performed on the resulting cDNAs using ABI StepOneTM Real-Time PCR Systems (Applied Biosystems, Foster City, CA, USA) [29]. The primer sequences utilized in this study are listed as follows: Nrf2 (NC_000002.12), 5′-CGGCTGGATTCTCCCCCAAG-3 and 5′-GTGATCCGACCGACCACG-3′; and HO-1 (NC_000022.11), 5′-CAGCAACAAAGTGCAAGGTGA-3′ and 5′-CCACCACCACCAAACATTCAG-3′ [31].

### 2.8. Western Blot

The Western blot analysis technique reported previously was utilized [29]. Primary antibodies targeting HGFs markers NRF2, HO-1 and NF-κB were employed from Cell Signaling Technology (Danvers, MA, USA). Secondary antibodies were employed from Santa Cruz Biotechnology (Dallas, TX, USA).

### 2.9. Enzyme-Linked Immunosorbent Assay (ELISA) Analysis

ELISA kits from R&D Systems (Minneapolis, MN, USA) were utilized to determine the concentrations of IL-6 and IL-8. Absorbance readings were measured using a 450 nm filter on a microplate reader (MRX; Dynatech Laboratories, Chantilly, VA, USA). Each sample was analyzed in triplicate [29].

### 2.10. Silencing of Nrf2 by Lentiviral-Mediated shRNAi

The pLV-RNAi vector was obtained from Biosettia Inc. (Biosettia, San Diego, CA, USA), with the protocol for replicating the double-stranded shRNA sequence provided in the manufacturer’s manuals [29,32]. In the establishment of a lentiviral expression vector, lentiviral vectors producing shRNA targeting human Nrf2 (NFE2L2) were generated and cloned into pLVRNAi. As an experimental control, a luciferase-targeting shRNA (sh-Luc: 5′-CCGGACTTACGCTGAGTACTTCGAACTCGAGTTCGAAGTACTCAGCGTA-3′) was utilized. Lentiviruses were produced by transfecting 293T cells with a combination of plasmid DNA, including the lentivector and helper plasmids (VSVG and Gag-Pol), using Lipofectamine 2000 (Invitrogen, Carlsbad, CA, USA). Supernatants were collected 48 h post-transfection and filtered, and virus titers were measured by FACS after an additional 48 h. Subconfluent cells were infected with lentivirus (Sigma-Aldrich) in the presence of 8 μg/mL polybrene. Green fluorescence protein (GFP), co-expressed in lentiviral-infected cells, served as a selection marker for identifying infected cells [29].

### 2.11. Statistical Analysis

Each experiment was conducted in triplicate. Statistical analysis was performed using one-way analysis of variance (ANOVA). Treatment differences were assessed using Duncan’s test, with a significance level set at *p* < 0.05.

## 3. Results

### 3.1. GMI Restored the Impaired Cell Proliferation in AGE/LPS-Stimulated HGF

**Figure 1:**
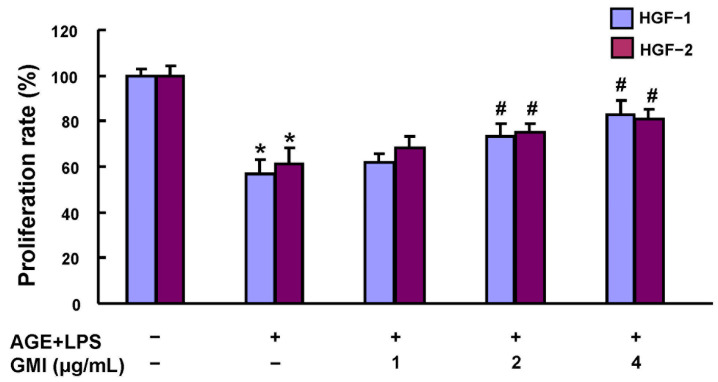
Effects of GMI on the cell proliferation rate in AGE/LPS-stimulated HGFs. The cell proliferation rate was measured by MTT assay. 1 × 104 cells/well of HGFs were seeded in 96-well plate with AGEs and LPS stimuli. GMI treatment of various dosages were given at the 24th hour, and the cells were further cultured for 48 h. Data represent the mean ± SD. * *p* < 0.05 compared to control group. # *p* < 0.05 compared to AGE/LPS group.

*Porphyromonas gingivalis* (*P. gingivalis*) is a key bacterium that has been strongly implicated in periodontitis development [33], where they elicit immune responses in periodontal tissues through its virulence factor, lipopolysaccharides (LPS) [34]. Meanwhile, AGEs are known to accumulate under chronic hyperglycemia [35] and have been observed particularly distributed in the highly inflamed gingiva in DP patients [36]. Thus, to simulate DP in vitro, we cultured HGFs with LPS from *P. gingivalis*. Subsequently, we assessed the impact of various dosages of GMI on the cell viability of these cells. The results showed that AGEs and LPS significantly inhibited cell proliferation, while GMI dose-dependently restored it (Figure 1).

### 3.2. AGE/LPS-Stimulated HGF Has Impaired Migration and Wound-Healing Capabilities While GMI Reversed These Phenomena

**Figure 2:**
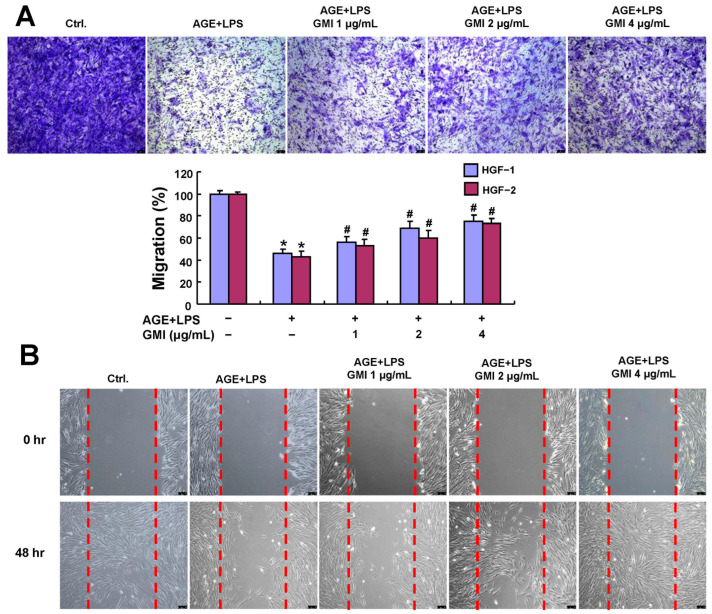
GMI improved the migration and wound-healing abilities in the AGE/LPS-stimulated HGFs. (**A**) Representative images and quantification data demonstrated that GMI treatment reversed the impaired migration ability in cells. (**B**) Wound-healing assay measured the cells’ wound-healing ability after GMI treatment from 0 to 48 h. The initial width of the wound created among the cells is represented by the red dashed line. Data were expressed in mean ± SD. Scale bars: 100 μm. * *p* < 0.05 compared to control group. # *p* < 0.05 compared to AGE/LPS group.

The buildup of cellular senescence, SASP and oxidative stress have been known to impair wound healing [15]. Therefore, we carried out migration and wound-healing assays among the AGE/LPS-stimulated HGFs to evaluate their ability to migrate and close the wound gap. Both assays demonstrated that HGF’s migratory and wound-healing abilities decreased tremendously when stimulated by AGEs and LPS. However, upon GMI treatment, these impaired features were markedly reversed (Figure 2A,B).

### 3.3. GMI Treatment Reduced ROS Production in AGE/LPS-Stimulated HGFs

**Figure 3:**
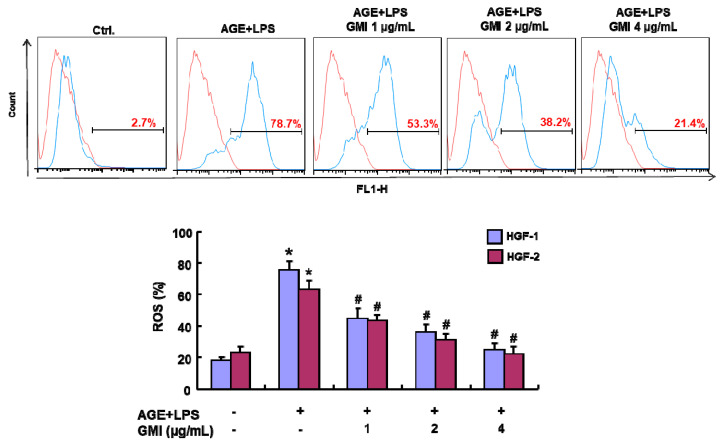
Effects of GMI on the production of ROS in the AGE/LPS-stimulated HGFs. DCFH-DA was used to assess the ROS activity in response to GMI treatments. The red line represent negative control, providing a baseline against which the fluorescence signals from stained samples (blue line) can be compared. AGE/LPS significantly upregulated the ROS production and this was suppressed upon GMI treatments in a dose-dependent manner. Data were expressed in mean ± SD. * *p* < 0.05 compared to control group. # *p* < 0.05 compared to AGE/LPS group.

Given the key players of inflammaging include oxidative stress, cellular senescence and SASP, we evaluated the effects of GMI on each of these parameters. For oxidative stress, we evaluated the impact of GMI on ROS production in AGE/LPS-induced HGFs. The results indicated that AGEs and LPS enhanced ROS secretions in HGFs, whereas GMI intervention repressed them in a dose-dependent manner (Figure 3).

### 3.4. GMI Inhibited Cell Senescence, SASP Secretions and NF-κB Phosphorylation in HGFs Subjected to AGE/LPS Stimuli

**Figure 4:**
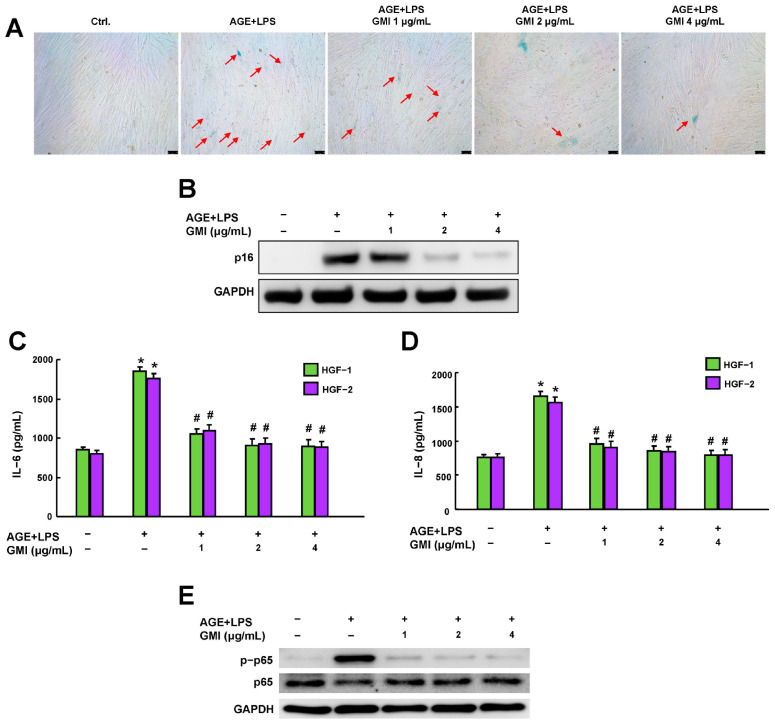
Effects of GMI on AGEs-induced cellular senescence, SASP and NF-κB phosphorylation. (**A**) Senescence-associated-β-galactosidase (SA-β-gal) assay showed an increase in SA-β-gal staining among the AGE+LPS cells, indicated by the red arrows, while addition of GMI suppressed them. (**B**) p16 expression level revealed the protective effects of GMI against the increased senescence activity. Additionally, GMI significantly reduced the secretions of (**C**) IL-6, (**D**) IL-8 and (**E**) phosphorylation of p65 in AGE/LPS-induced HGFs. * *p* < 0.05 compared to control group. # *p* < 0.05 compared to AGE/LPS group.

Next, we explored the effects of GMI on the activities of cellular senescence and SASP secretions such as IL-6 and IL-8 [8,9,10,11,12] as well as the expression of nuclear factor-κB (NF-κB), a key regulator of these cytokines [37]. In the AGE/LPS-induced cells, markers of cell senescence, including SA-β-Gal staining and p16 expression (Figure 4A,B and Figure S1), were upregulated. Additionally, there was an increase in IL-6, IL-8 secretion and NF-κB phosphorylation (Figure 4C–E and Figure S2). Meanwhile, GMI intervention significantly inhibited the heightened cell senescence expression, IL-6 and IL-8 secretions, and NF-κB phosphorylation (Figure 4).

### 3.5. GMI Upregulated the Nrf2/HO-1 Pathway in AGE/LPS-Stimulated HGFs

**Figure 5:**
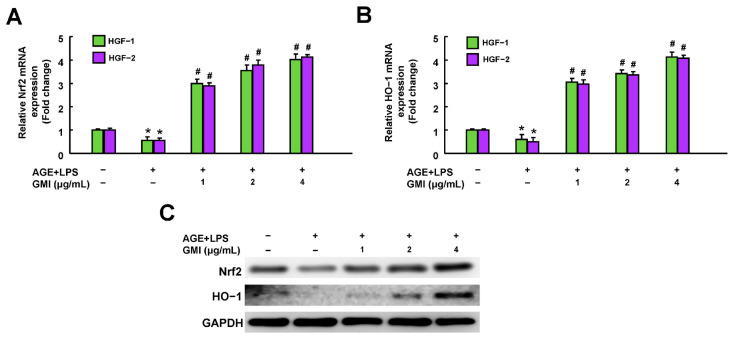
The expressions of mRNA and protein of Nrf2 and HO-1 in AGE/LPS-stimulated HGFs after GMI treatment. In a dose-dependent manner, GMI treatment increased the relative mRNA (**A**,**B**) and protein expressions (**C**) of Nrf2 and HO-1 in AGE/LPS-stimulated HGFs. Data represent the mean ± SD. * *p* < 0.05 compared to control group. # *p* < 0.05 compared to AGE/LPS group.

Given that Ganoderma lucidum has the ability to modulate the Nrf2 pathway [23,24], we elucidated if GMI regulates AGE/LPS-stimulated inflammaging in DP via the Nrf2/HO-1 signaling pathway. We first analyzed the effects of GMI on the mRNA and protein expressions of Nrf2 and HO-1 among the cells. It was found that AGEs decreased the mRNA (Figure 5A,B) and protein expressions (Figure 5C and Figure S3) of Nrf2 (NFE2L2) and HO-1 (HMOX2) in these cells. On the other hand, treatment with GMI reversed these phenomena in a dose-dependent manner, even surpassing the control group (Figure 5A–C).

### 3.6. GMI’s Anti-Inflammaging Trait Was Exerted via the Nrf2 Signaling Pathway

**Figure 6:**
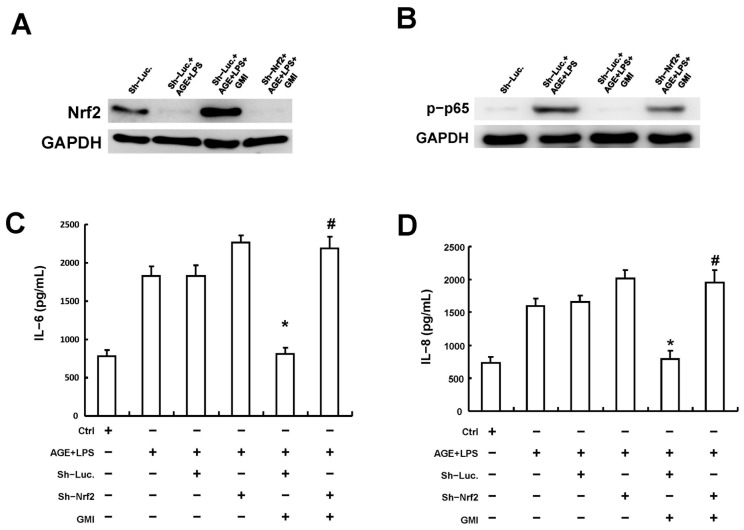
GMI’s repression on both p65 phosphorylation and secretions of IL-6 and IL-8 were reversed upon the silencing of Nrf2. (**A**) Western blotting analysis revealed the effectiveness of Nrf2 knockdown in AGE/LPS-stimulated HGFs. The expression of p65 phosphorylation (**B**) and levels of IL-6 (**C**) and IL-8 (**D**) were measured in AGE/LPS-stimulated HGFs with and without sh-Nrf2. * *p* < 0.05 compared to sh-Luc group, # *p* < 0.05 compared to AGE/LPS + GMI group.

**Figure 7:**
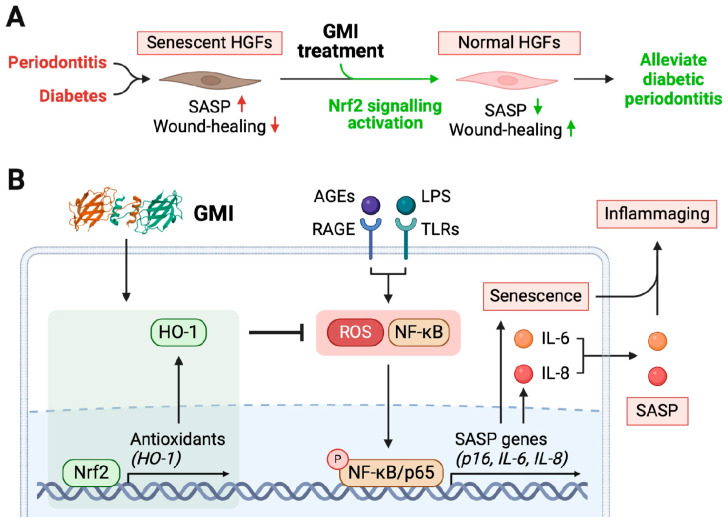
GMI treatment alleviates inflammaging in diabetes-associated periodontitis through the activation of the Nrf2 pathway. (**A**) Both periodontitis and diabetes stimuli result in cellular senescence and the development of SASP in HGFs. These lead to hindered wound healing and persistent inflammation of the periodontal tissue. Conversely, GMI treatment can inhibit HGFs from undergoing cell senescence by activating the Nrf2 signaling pathway. GMI decreases SASP, improves wound healing, and ultimately alleviates diabetic periodontitis. (**B**) The diagram illustrates that GMI treatment increases the expressions of Nrf2 and HO-1 in cells, which subsequently inhibit the rise in ROS and NF-κB pathway. Surge of intracellular ROS and NF-κB activation are known to be induced by AGEs and LPS from *P. gingivalis* through RAGE and TLRs, respectively. Elevated ROS and active NF-κB lead to the expression of the senescence gene (e.g., p16) and pro-inflammatory cytokines (e.g., IL-6 and IL-8), resulting in elevated cellular senescence and SASP phenotype. These ultimately lead to inflammaging and impair the wound-healing capacity of HGFs. On the other hand, the addition of GMI subsequently inhibits the SASP of HGFs, reduce tissue inflammaging and restore wound-healing capacity in DP. This could be a promising new approach for alleviating periodontitis associated with diabetes. HGFs, human gingival fibroblasts; SASP, senescence-associated secretory phenotype; GMI, *Ganoderma microsporum* immunomodulatory protein; Nrf2, nuclear factor-erythroid 2 (NF-E2)-related factor 2; HO-1, heme oxygenase-1; AGEs, advanced glycation end-products; RAGE, receptor for advanced glycation end-products; LPS, lipopolysaccharide; TLRs, Toll-like receptors; ROS, reactive oxygen species; NF-κB, nuclear factor kappa B subunit 1; IL, interleukins. The three-dimensional structure of GMI is sourced from the RCSB Protein Data Bank (https://www.rcsb.org, accessed on 30 May 2024).

Once we verified the involvement of GMI in the upregulation of Nrf2/HO-1 among the AGE/LPS-stimulated HGFs, we proceeded to verify if GMI exerts its anti-inflammaging trait via the Nrf2 signaling pathway. Thus, Nrf2 was first knocked down in HGFs, followed by the assessments of p65 phosphorylation and SASP secretions, IL-6 and IL-8. The Western blotting analysis showed that Nrf2 knockdown was efficiently carried out in the AGE/LPS-stimulated HGFs (Figure 6A and Figure S4). The protective effects of GMI against p65 phosphorylation (Figure 6B and Figure S5) and IL-6 and IL-8 secretions (Figure 6C,D) were reversed upon the knockdown of Nrf2, suggesting that GMI exerts its anti-inflammaging effect via the upregulation of the Nrf2 signaling pathway (Figure 7).

## 4. Discussion

It is well-established that the bidirectional impact of diabetes mellitus and periodontitis exacerbates local and systemic disease progression [2,3,4]. Persistent hyperglycemia in diabetic patients has been shown to promote inflammaging, a state characterized by chronic inflammation with aging, which impairs tissue homeostasis and leads to poor wound healing [7,9,19,20,38,39]. A cross-sectional study reported that patients with diabetes-associated periodontitis (DP) had elevated levels of senescence-associated secretory phenotype (SASP) factors and aging markers in their serum and gingival tissues, significantly correlating with the severity of periodontitis [13]. Interestingly, while human gingival fibroblasts (HGFs) with SASP can be obtained from patients’ periodontitis tissues, short-term stimulation with LPS was insufficient to induce SASP of HGFs in vitro [40]. These findings may imply that the presence of diabetes creates conditions more conducive to cellular aging, thereby accelerating periodontitis development. Our previous studies confirmed that AGEs-alone treatment was sufficient to induce the activation of NF-κB (phosphorylation of p65) and SASP of primary HGFs, as well as the impairment of cell proliferation and migration [26,41,42,43,44]. Compared to these results, this study found additive effects of combined treatment with AGEs and LPS (AGEs/LPS), which induced greater activity of NF-κB and higher expression of senescence markers (p16, Figure 4). These results support the use of AGE/LPS co-treatment as a more appropriate method for studying the pathological mechanisms of periodontitis with diabetes. Our works also showed that Ganoderma immunomodulatory protein (GMI) reversed the adverse effects of AGEs/LPS on cell proliferation, cell migration, and SASP of HGFs, suggesting that GMI exhibits anti-inflammaging properties under DP conditions.

The NF-κB pathway is a classical downstream target of AGEs/RAGE- and LPS-induced signaling [45,46]. Indeed, gingival tissue from the DP group had higher levels of RAGE and NF-κB mRNA compared to tissues from healthy individuals and those with periodontitis alone [47]. Inhibition of NF-κB activation, either pharmacologically or by transgenic methods, improves bone resorption in experimental DP in vivo [48,49]. Moreover, in vitro evidence suggests that AGEs/RAGE signaling induced IL-6 secretion of HGFs primarily via activating NF-κB [48]. In line with these results, we discovered that GMI treatment resulted in a remarkable shielding of HGFs against AGE/LPS-induced NF-κB activation (p-p65), IL-6 and IL-8 secretion, and cellular senescence in a dose-dependent manner (Figure 4). These findings indicate that GMI reversed AGE/LPS-induced inflammaging by negatively regulating NF-κB, which is consistent with the findings reported in other studies [26,27]. However, the mechanism by which GMI inhibits the release of pro-inflammatory factors may be context-dependent. For instance, GMI treatment was shown to activate the STAT3/IL-6 axis of C2C12 cells, which is crucial for myogenesis [50]. Thus, we propose that GMI exerts anti-inflammatory effects related to the NF-κB activation in a persistent inflammatory condition.

Studies have found that patients with higher levels of oxidative damage markers in a gingival crevicular fluid are more likely to develop severe periodontitis [51]. Nuclear factor-erythroid 2-related factor 2 (Nrf2) pathway, such as Nrf2/heme oxygenase-1 (HO-1) axis, plays a central role in cellular defense mechanisms against oxidative stress [52], directly activating Nrf2 or preventing its degradation can mitigate alveolar bone loss in vivo [53,54,55]. Therefore, maintaining oxidative stress homeostasis is crucial for periodontium tissue health. In addition, a recent study has shown that HGFs derived from periodontitis tissues displayed an increased SASP, which can be reversed by treating with N-acetylcysteine (a ROS scavenger) [56]. This suggests that elevated oxidative stress is a key event allowing the induction of SASP in periodontitis. Indeed, quercetin, a senolytic agent, can directly bind to Nrf2 to suppress NF-κB-mediated SASP in inflammatory nucleus pulposus cells and ameliorate the experimental intervertebral disc degeneration [57]. Notably, activation of AGEs/RAGE triggered the NF-κB pathway regulated by AGEs/RAGE-induced oxidative stress [58]. It may imply that the activation of Nrf2, at least in part, acts as an upstream mechanism that negatively regulates NF-κB-mediated SASP under AGE stimulation. In line with this, our findings demonstrated that GMI intervention reversed the increased cellular ROS and the suppression of both gene and protein expressions of Nrf2 and HO-1 induced by AGEs/LPS in a dose-dependent manner (Figure 4 and Figure 5). Importantly, GMI treatment failed to suppress the AGE/LPS-induced activation of NF-κB (Figure 6B) and the release of IL-6 and IL-8 of HGFs when the Nrf2 was silenced (Figure 6C,D), suggesting that GMI impedes NF-κB activation and SASP by increasing Nrf2 rather than directly inhibiting NF-κB. Overall, this study is the first to provide evidence that GMI regulates the Nrf2/NF-κB axis and inhibits the subsequent SASP in the context of DP.

Periodontitis is a local inflammatory disease caused by pathogenic bacteria [1]. Growing evidence shows that virulence factors produced by these microbiotas could lead to islet β-cell dysfunction and insulin resistance [59]. Therefore, GMI intervention might not only improve periodontitis but also help manage diabetes development. Additionally, considering the anti-inflammaging properties of GMI, it may offer new perspectives for managing or improving periodontium health in elderly patients, as well as protecting against other aging-related periodontitis comorbidities [60]. Furthermore, patients with refractory periodontitis who are susceptible to producing ROS exhibit greater severity of periodontitis [61]. This suggests that such patients may be more sensitive to GMI intervention due to its anti-inflammaging effects through preferential activation of Nrf2. Altogether, we propose GMI holds promising clinical potential, offering a valuable direction for future research.

The progression of periodontitis results from a dynamic microenvironment shaped by cell-cell and cell-microbe interactions [1,59], which may be more complex in the context of diabetes [13,49,53,55,62,63,64,65,66]. For instance, the biological behavior of infiltrating pro-inflammatory immune cells and the composition of the subgingival microbiome are significantly altered in diabetes-associated periodontitis [49,55,64,65,66]. In experimental DP mice, it was found that the receptor activator of nuclear factor kappa-Β ligand (RANKL) was primarily expressed in periodontal ligament fibroblasts rather than in HGFs [49]. This highlights that the function of fibroblasts is influenced by their spatial distribution within periodontal tissues. While the study suggests positive effects of GMI on AGE/LPS-stimulated HGFs, its applicability to understanding GMI treatment for DP progression is currently limited. Thus, to advance our understanding, future research should utilize co-culture in vitro systems and experimental DP animal models. These approaches will better evaluate the therapeutic potential of GMI intervention. Additionally, conducting molecular docking analysis is crucial to explore the precise mechanism through which GMI activates Nrf2.

## 5. Conclusions

In conclusion, GMI treatment effectively suppresses inflammaging and improves wound healing in diabetic periodontitis models in vitro. It notably reversed AGE/LPS-induced inflammaging components in HGFs, including increased oxidative stress, cellular senescence and levels of IL-6 and IL-8. These anti-inflammaging effects are likely to be mediated through the regulation of the Nrf2/NF-κB axis, suggesting a potential novel therapy for periodontitis in diabetic patients (Figure 7). Further in vivo studies are warranted to validate these findings and explore clinical implications.

## Data Availability

All data are included within the article.

## Supplementary Materials

The following supporting information can be downloaded at: https://www.mdpi.com/article/10.3390/antiox13070817/s1, Figure S1. Original immunoblotting data for Figure 4B; Figure S2. Original immunoblotting data for Figure 4C; Figure S3. Original immunoblotting data for Figure 5C; Figure S4. Original immunoblotting data for Figure 6A; Figure S5. Original immunoblotting data for Figure 6A.
