# Supplementary material for: Ganoderma Microsporum Immunomodulatory Protein Alleviates Inflammaging and Oxidative Stress in Diabetes-Associated Periodontitis via Nrf2 Signaling Activation: An In Vitro Study
Source: Antioxidants (Basel). 2024 Jul 8;13(7):817. doi: 10.3390/antiox13070817 (PMC11273761; doi:10.3390/antiox13070817)
Supplement: Supplementary file 1 [file antioxidants-13-00817-s001.zip › antioxidants-3070596-supplementary.pdf]

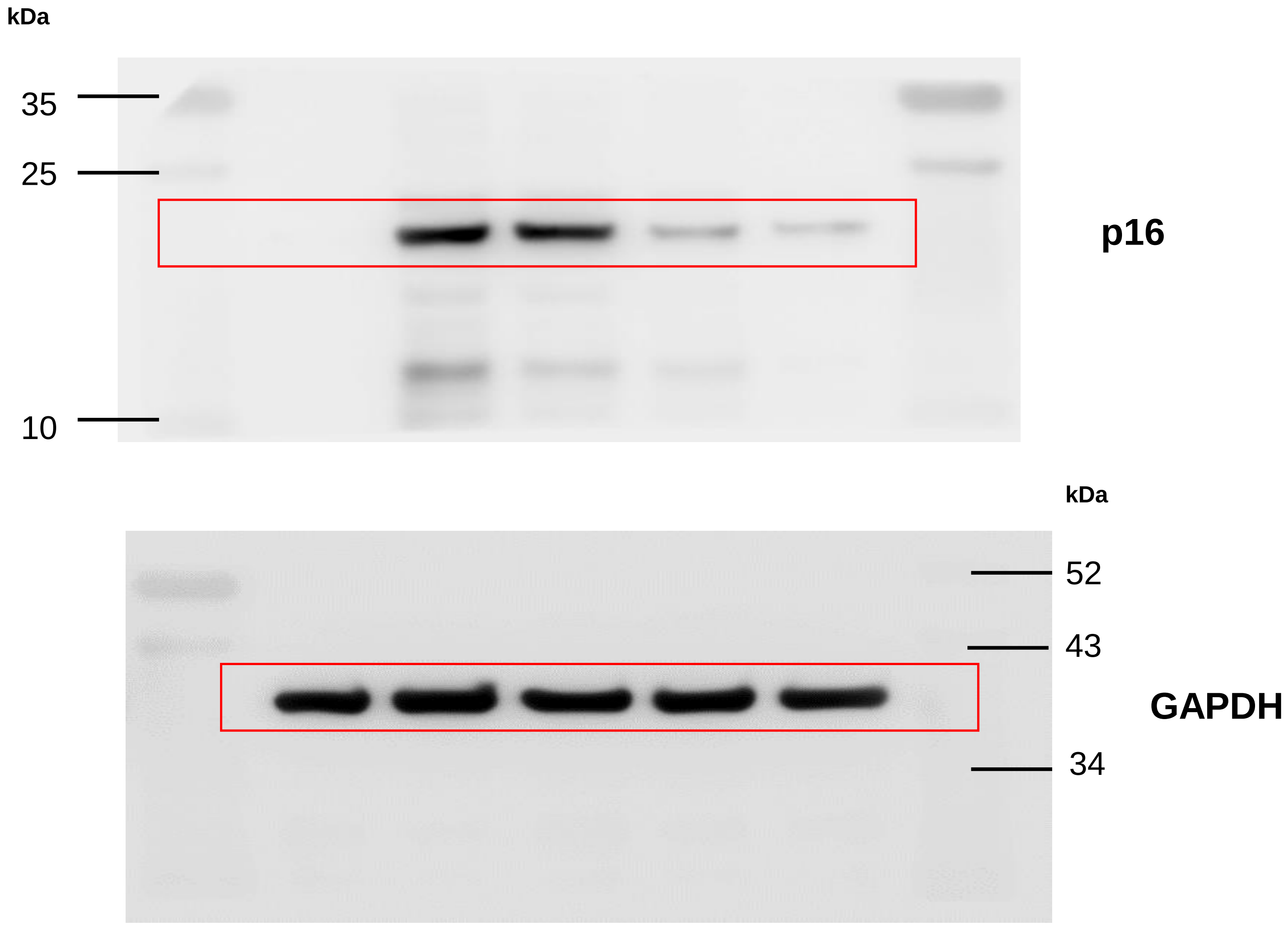

Supplementary Figure S1. Original immunoblotting data for Fig. 4B

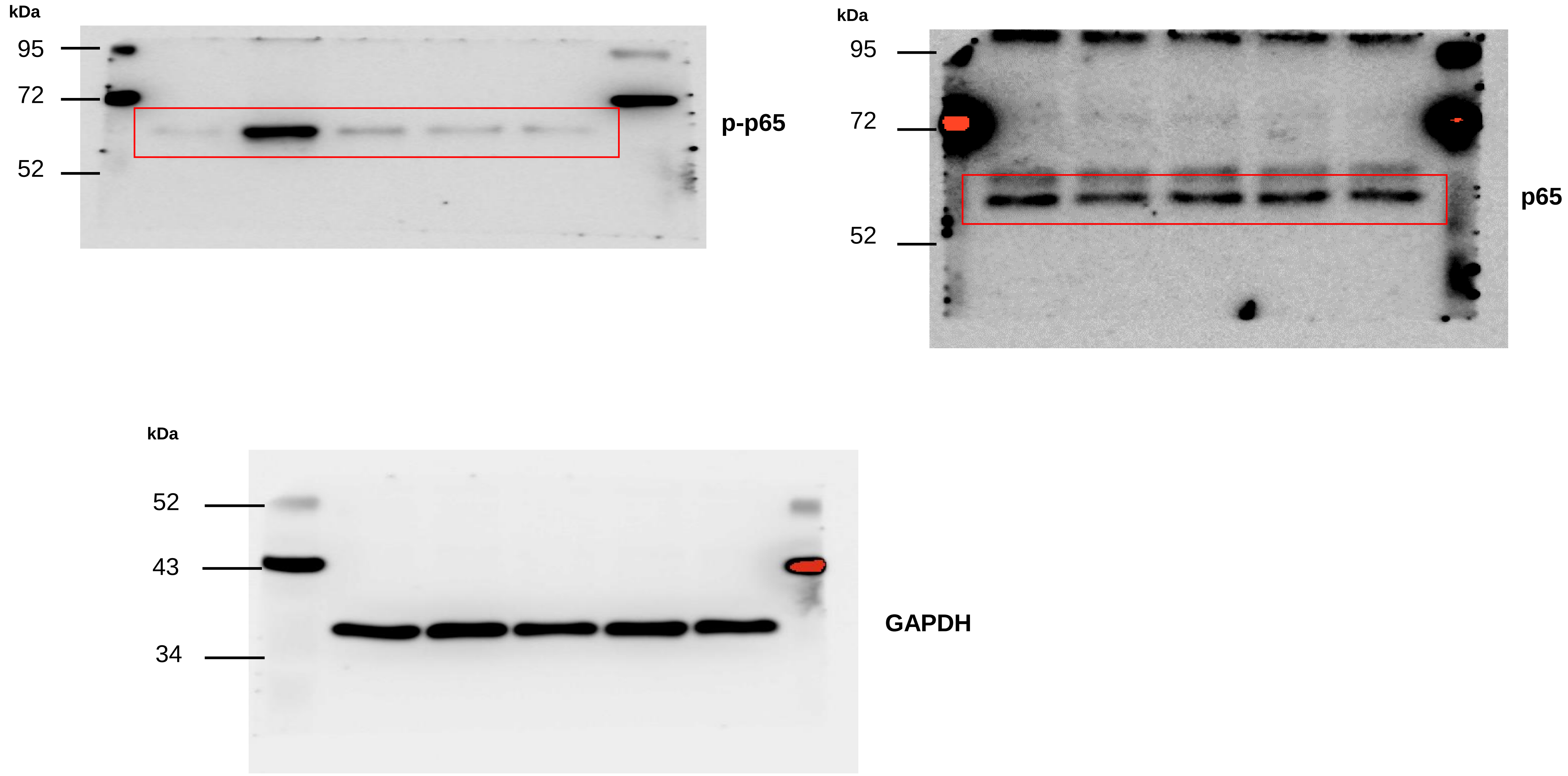

Supplementary Figure S2. Original immunoblotting data for Fig. 4C

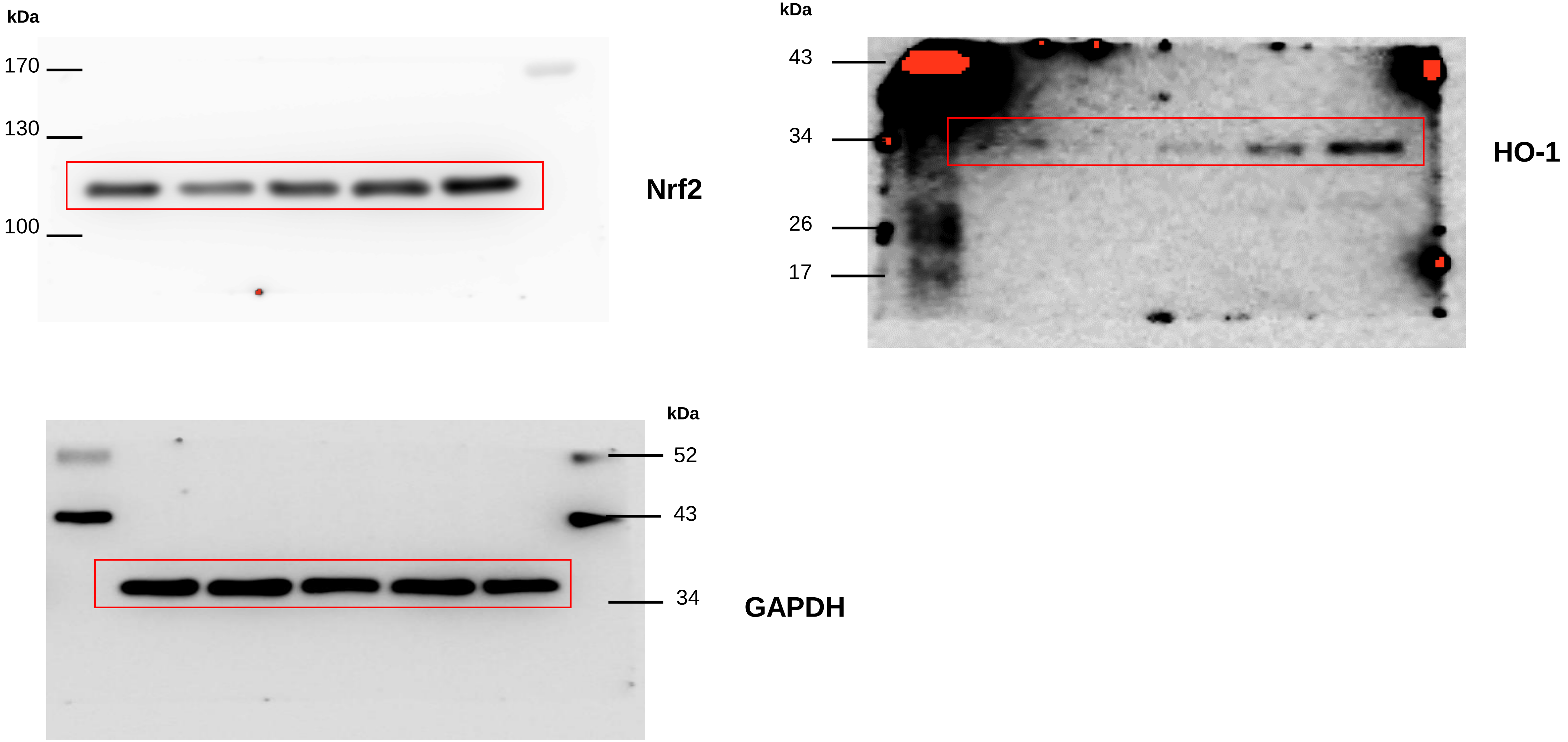

Supplementary Figure S3. Original immunoblotting data for Fig. 5C

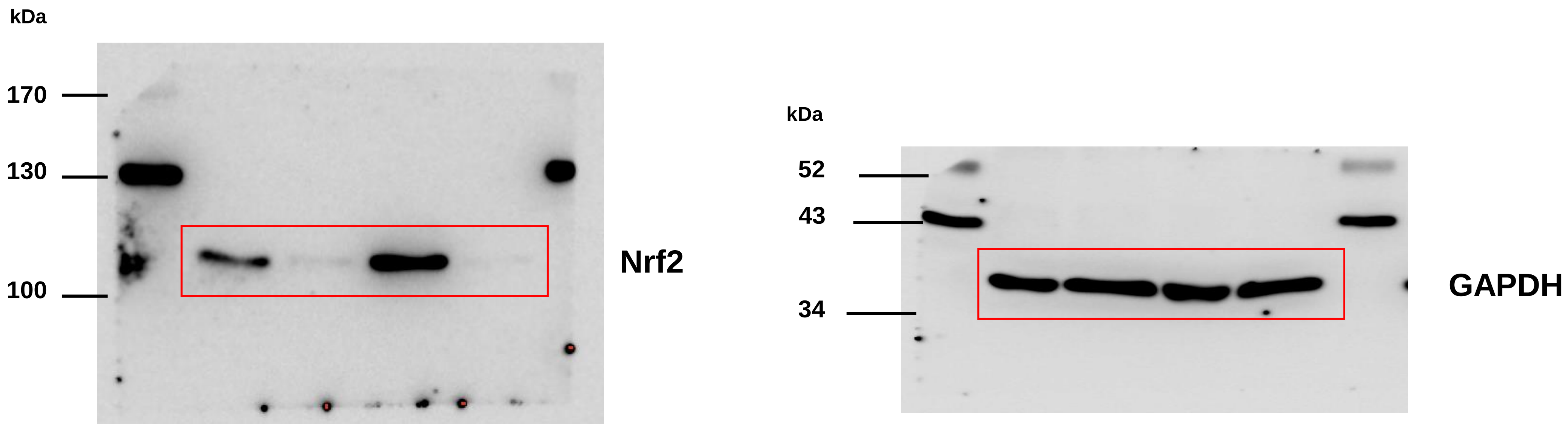

Supplementary Figure S4. Original immunoblotting data for Fig. 6A

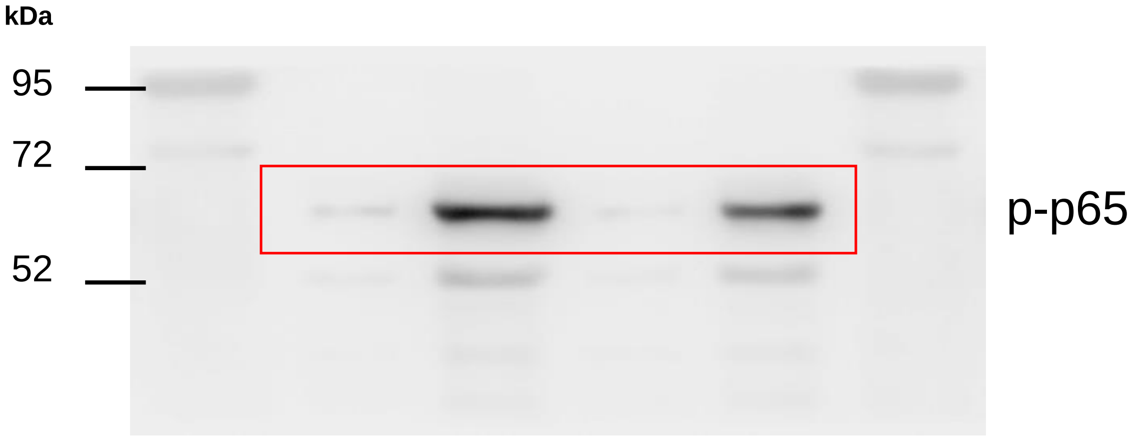

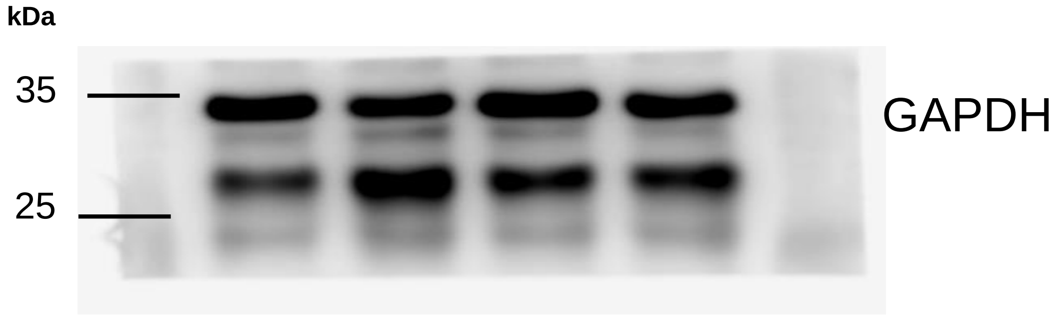

Supplementary Figure S5. Original immunoblotting data for Fig. 6A
